# Method for 3D Airway Topology Extraction

**DOI:** 10.1155/2015/127010

**Published:** 2015-02-12

**Authors:** Roman Grothausmann, Manuela Kellner, Marko Heidrich, Raoul-Amadeus Lorbeer, Tammo Ripken, Heiko Meyer, Mark P. Kuehnel, Matthias Ochs, Bodo Rosenhahn

**Affiliations:** ^1^Institute of Functional and Applied Anatomy, Hannover Medical School, Hannover, Germany; ^2^REBIRTH Cluster of Excellence, Hannover, Germany; ^3^Biomedical Research in Endstage and Obstructive Lung Disease Hannover (BREATH), Member of the German Center for Lung Research (DZL), Hannover, Germany; ^4^Biomedical Optics Department, Laser Zentrum Hannover e.V., Hannover, Germany; ^5^Department of Cardiothoracic, Transplantation and Vascular Surgery (HTTG), Hannover Medical School, Hannover, Germany; ^6^Institut für Informationsverarbeitung, Leibniz University Hannover, Hannover, Germany

## Abstract

In lungs the number of conducting airway generations as well as bifurcation patterns varies across species and shows specific characteristics relating to illnesses or gene variations. A method to characterize the topology of the mouse airway tree using scanning laser optical tomography (SLOT) tomograms is presented in this paper. It is used to test discrimination between two types of mice based on detected differences in their conducting airway pattern. Based on segmentations of the airways in these tomograms, the main spanning tree of the volume skeleton is computed. The resulting graph structure is used to distinguish between wild type and surfactant protein (SP-D) deficient knock-out mice.

## 1. Introduction

The functional capacity of the mammalian lung is reflected in its architecture. Air is conducted to the alveolar region where gas exchange takes place over a large surface across a thin air-blood barrier. The conducting airways are arranged as a branching tree, starting outside the lung with the trachea which bifurcates into the right and left main bronchi which then enter the right and left lung, respectively, where they further subdivide [1]. The number of conducting airway generations as well as bifurcation patterns varies across species and shows specific characteristics, for example, when comparing rodents and primates [2].

Nondestructive imaging techniques allow for 3D visualization and quantitative characterization of intrapulmonary conducting airways [3–5]. One promising technique that was recently made available for ex vivo imaging of fixed mouse lungs is scanning laser optical tomography (SLOT) [6]. SLOT is a highly efficient laser-based microscopy technique allowing rapid scanning of the whole mouse lungs. Based on intrinsic contrast (fluorescent and nonfluorescent signals), projection images are recorded while rotating the sample enabling 3D tomographic reconstruction [7, 8].

One advantage of the use of mouse models of lung diseases in pulmonary research is the ability to perform gene manipulations in order to identify pathophysiological pathways and potential therapeutic targets which can then be tested in further studies. One example is the complex phenotype of mice deficient in surfactant protein D (SP-D). These SP-D knock-out mice develop intra-alveolar accumulation of surfactant material (lipoproteinosis) and alterations of surfactant-producing alveolar epithelial type II cells as well as emphysematous alterations associated with an increased baseline inflammatory level that is suspected to contribute to the destructive lung remodelling seen in these mice [9–11].

In this study an image analysis method to characterize the topology of the mouse airway tree using SLOT datasets is presented. To demonstrate the feasibility of this approach and its potential value, a set of mice, part of which deficient for SP-D, was used to see whether the analysis was able to discriminate between wild type and SP-D knock-out mice based on detected differences in their conducting airway pattern.

**Figure 1:**
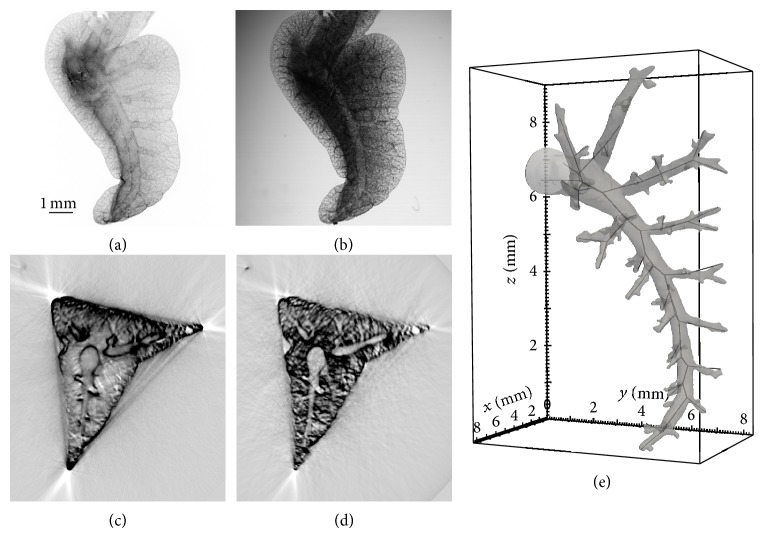
SLOT projection datasets: fluorescence image of the PMT (a) and absorption image of the PD (b), slices from the tomograms: (c), (d) accordingly, (e) segmented airways (transparent grey) and computed main skeleton (black).

The method presented is based on the computation of approximate skeletons representing the airway tree topologies (airways, junctions, and endpoints) as visualized in Figure 1. 3D segmented regions of the airways in the SLOT tomograms (an example shown in transparent grey) are used as input. The computed skeleton used for further lung analysis is included as a black graph.

The proposed analysis of the airway tree topology has several advantages.

- The derived features are rotation, translation, and scale invariant.
- The features are invariant with respect to deformations during sample preparation.
- The features can be derived from different imaging modalities. It allows for an imaging-independent analysis, as long as the airway can be extracted.
- Our method gives for the first time the indication that airway topology and SP-D deficiency (usually only affecting the alveolae) are correlated.

## 2. Sample Preparation, Data Acquisition, and Segmentation

A total of seven mouse lungs were analysed in this study. The lungs were taken from C57BL/6 wild type (*n* = 4) as well as SP-D deficient (*n* = 3) mice [12, 13]. Mice were housed in the animal facility of the Hannover Medical School, with food and water provided ad libitum. All animals were treated in compliance with the* Principles of Laboratory Animal Care* formulated by the National Society for Medical Research and the* Guide for the Care and Use of Laboratory Animals*, published by the National Institute of Health (NIH publication 85-23, revised in 1996), as well as in compliance with the Protection of Animals Act, approved by the bioethical committee of the district of Lower Saxony.

### 2.1. Lung Processing

Preparation of the lungs was performed as described in Kellner et al. [6]. Briefly, the lungs were fixed in situ by intravascular perfusion of a mixture of 0.1% glutaraldehyde and 4% formaldehyde (from freshly depolymerized paraformaldehyde) in 0.2 M Hepes buffer [14]. After anesthesia, the lungs were inflated via tracheal intubation using a pressure of 13 cm liquid column. Subsequently the abdomen was opened and the inferior caval vein was cannulated. After a short preflush, perfusion fixation was performed at a pressure of 30 cm liquid column.

### 2.2. Data Acquisition

In order to obtain SLOT datasets, the lungs were dehydrated in an increasing ethanol series of 30, 50, 70, 90, and 99.8% ethanol with an incubation time of at least 2 hours per ethanol step. The lungs were then transferred into a mixture of 3 parts methylsalicylic acid (MS) and 2 parts benzyl benzoate (BB) for clearing. Transparency of the lungs was reached after 2-3 hours of treatment. For SLOT imaging, the accessory lobe was prepared and fixed with a cannula upside down in the SLOT imaging chamber. For image acquisition the sample was rotated stepwise 360° to acquire projection images using 532 nm excitation combined with a 570 nm optical long pass filter. Both, absorption (Photo Diode PD) and fluorescence (Photo MulTiplier PMT) images (Figure 1) of 700 projection directions were used to reconstruct 3D representations of the sample (tomograms) employing a back projection algorithm.

### 2.3. Data Preprocessing and Segmentation

The tomograms obtained from transmission datasets were multiplied with the corresponding fluorescence tomograms in order to reduce noise and to enhance the contrast originating from airway tissue using Fiji [15]. The* adaptive brush* (a tool available in 2D or 3D) was used to segment the lumen of the airways of the resulting tomograms in ITKsnapm [16] [http://www.itksnap.org/] building on ITK [17] [http://www.itk.org] and VTK [18] [http://www.vtk.org] functions. This semiautomatic segmentation process employs a gradient-anisotropic-diffusion image filter, a gradient-magnitude image filter, and a watershed transform. Only the label under the cursor is used from the result and assigned to the segmentation of the airway lumen. The final segmentation was convolved with an uncertainty kernel of around 3 voxel to account for the estimated measurement error. Only voxels belonging to the segment with a probability of at least 50% were regarded. This leads to smoothed surfaces generated with the marching cubes algorithm from VTK rendered with ParaView [19] [http://www.paraview.org].

## 3. Image Analysis Methods

The method to extract the prominent topology of the airways is based on two basic algorithms: First a 3D skeleton is computed and then dominant information is derived.

### 3.1. 3D Skeleton Computation

**Figure 2:**
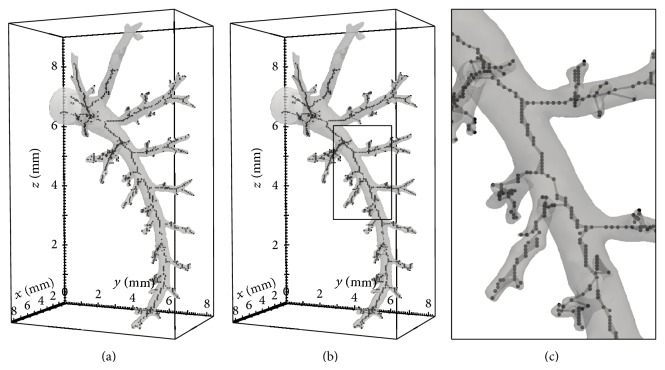
(a) Skeleton consisting of disconnected points after 2D skeletonization. (b) Point connections created by a minimal spanning tree computation. (c) The close-up visualizes the inherent problems of the resulting skeleton tree.

A common procedure to compute 3D skeletons is based on the voxel-wise multiplication of 2D skeletons computed through all volume slices along the *x*-, *y*- and *z*-axes. Since the outcome is usually a disconnected binary volume (see Figure 2(a)), a minimal spanning tree [20] can be used to connect the 3D points to ensure a 3D connected tree (see Figures 2(b) and 2(c)). In the present method, the minimal spanning trees are computed with Prim's algorithm which can be summarized as follows. Initialize a tree with a single vertex, chosen arbitrarily from the graph. Grow the tree by one edge, find the minimum-weight edge of the edges that connect the tree to vertices not yet in the tree, and transfer it to the tree. Repeat until all vertices are in the tree.

A problem of 3D skeleton extraction consists of noise artefacts, usually on endpoints and dilated areas of the airways, as shown in the close-up of Figure 2. Therefore, the extracted skeleton cannot be used in this form, since the dominant information is overruled by the artefacts in the data.

### 3.2. Dominant Skeleton Computation

The key idea for extracting the dominant graph structure behind the 3D skeleton is outlined as follows.

**Figure 3:**
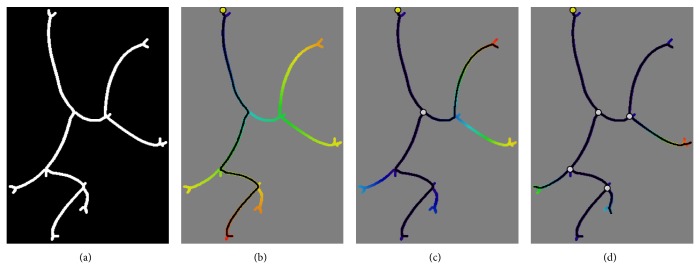
Exemplary images visualizing different stages of the analysis. (a) Exemplary 2D binary image. (b) The distance transform (distance from seed points colour-coded from blue to red) computed on the binary image starting from the seed point (yellow) and the resulting longest path (black). (c) Second computed distance transform starting from the first path and the resulting second path connected to the first one (white circle). (d) Final tree structure, ignoring any further small branches.

First a distance transform from a start point (e.g., an entrance point at the start of the airway, which can easily be identified, yellow point in Figure 3) along the skeleton is computed. Then the furthest resulting distance point is selected as end point. This is the first leaf of the resulting skeleton. Then the path is backtracked as shown in Figure 3(b). Secondly, starting from the backtracked path, another distance transform is computed to identify the second largest branch along the airway and backtracked analogously.

As the full set of points on the first trajectory (not just a starting point) are used for distance computation, there is no overlap between the first and second path, just a connection point. This is shown in Figure 3(c). The intersection between the first and the second path yields the first junction of the resulting skeleton tree, marked with a white point. This procedure is repeated until a local distance measure is minimized. The minimal distance is chosen such that only the prominent branches are extracted and short leaves likely originating from noise are suppressed (Figure 3(d)).

**Figure 4:**
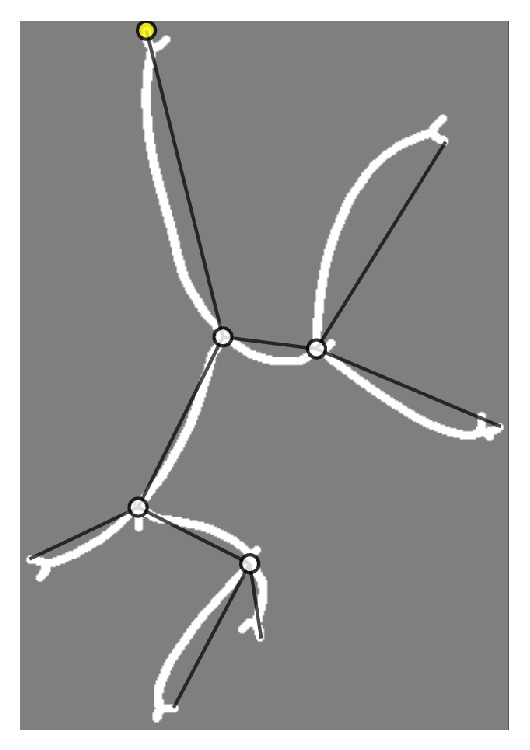
Final topological graph.

Based on the junctions, end points and connections of the topological graph can easily be extracted, as shown in Figure 4.

### 3.3. Exemplary Application

**Figure 5:**
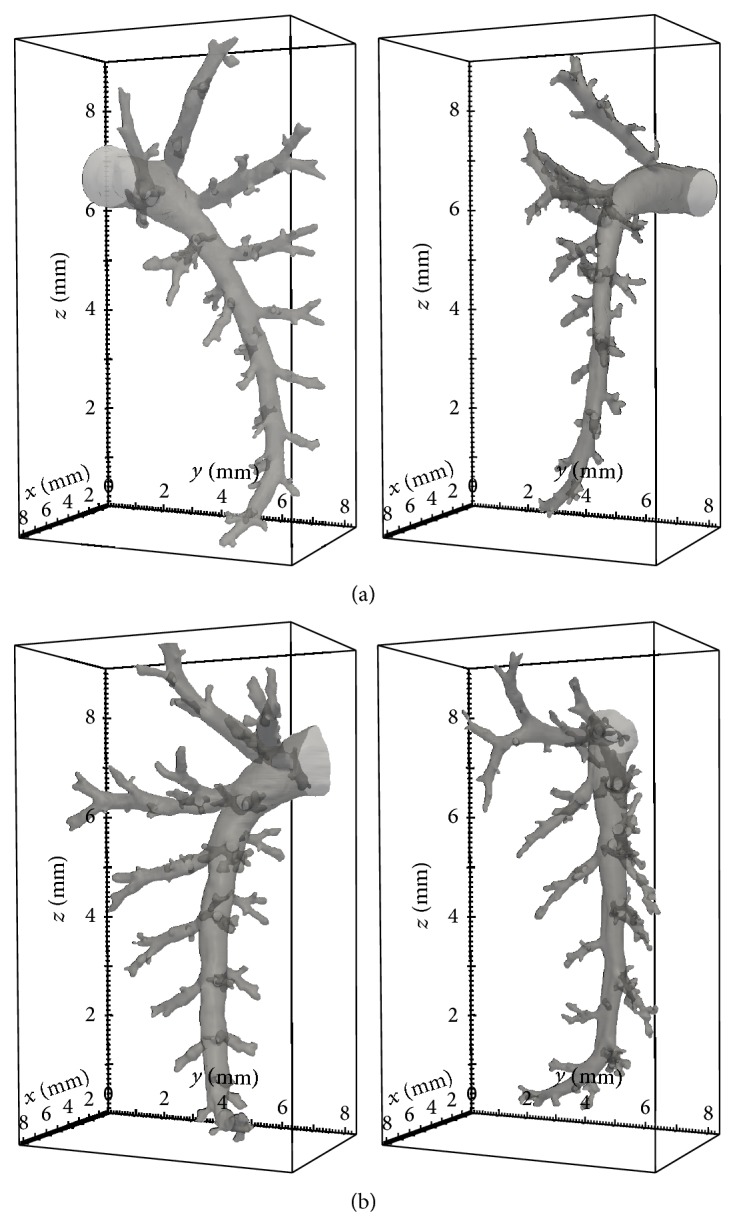
Examplary airways ((a) wild type, (b) SP-D deficient) used for the classification demonstration. Visually no difference can be seen.

**Figure 6:**
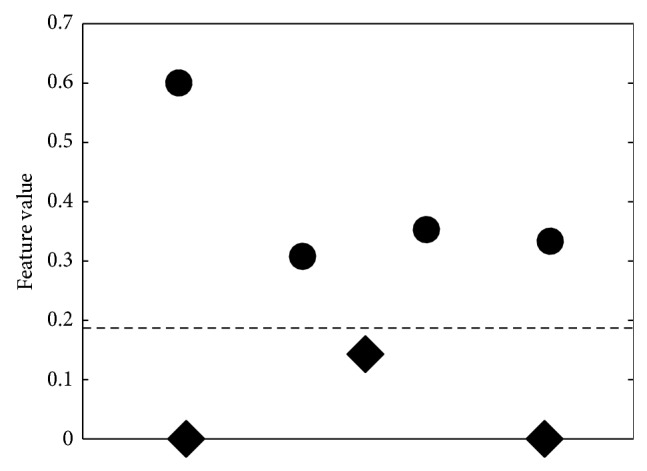
Feature values for a junction degree of 4 as used for classification. Circles mark wild type and diamonds SP-D. A threshold (derived with a Bayes classifier) can perfectly split the data (dashed black line).

Dataset of the seven tomograms obtained by SLOT as described in Section 2 are used for topological airway analyses. Some examples are shown in Figure 5. After the extraction of the topology graphs (Section 3.2), prominent features are chosen for a cluster analysis. For example, the amount of 3, 4, or 5 junctions can be used as features (Table 1). Note, if we have a junction and one branch splits from the main airway, it is called a three-junction since at the junction point itself there are three parts branching, two along the main branch and one sideway. Two branching paths along the main branch form a 4-junction, which is the interesting case more frequently occurring in SP-D deficient mice. The only parameter involved is a threshold to merge close junctions across the dominant skeleton. This parameter has been determined empirically.  Figure 6 shows the amount of 4 junctions of each dataset, summed up approx. 75% along the largest airway. The values obtained for the wild type datasets are drawn in blue and for the SP-D deficient mice in red. It can be seen that two clusters exist and a threshold obtained by a trained Bayes classifier [21] (shown in black) can perfectly split the datasets.

Additionally, a leave-one-out experiment was performed, using six datasets for training a Bayes classifier and applying it to the remaining dataset. The confusion table (Table 2) shows that only the classification of one SP-D sample was wrong.

## 4. Discussion

Most work concerning classification of image data of healthy versus diseased lungs is based on X-ray computed tomography (CT) scans, most but not all making use of the 3D relations [22–26]. For example, CT scans of the same lungs at different inflation states can be discriminated from other lung datasets by an automated method described by Feragen et al. [23]. The method is based on comparisons of geodesic and topological changes of skeletons of airway trees. Lung diseases can also be classified directly on CT tomograms employing statistical or integral geometric and topological measures (like gray-level occurrence matrices, Minkowski dimensions, and Minkowski Functionals) on lung tissue textures avoiding the generation of airway tree segmentation/skeletons [24–26]. Skeletons often are created from binary images, which implies a preceding segmentation of the airway lumen, that is, classification into foreground and background. Both steps can introduce errors. Therefore, many attempts have been made to improve segmentation methods, some are specific for airways specialized for, for example, in vivo CT scans taking surrounding blood vessel configurations into account [22, 27, 28]. Skeletons can also be computed directly on grey images without the need for preceding segmentations [29, 30] or in combination [27, 31].

Despite the fact that in the present study mouse lungs imaged with SLOT were used for the classification based on airway tree skeletons, a feature like the used relative number of branching level (i.e., *n*-junction) seems uncommon so far. Its description of the topology of airway trees is more specific than other measures such as, for example, the Euler–Poincaré characteristic. In contrast to grey-level analyses and classifications it is less dependent on local variations and far lighter concerning computation dimensions. Therefore, however, it cannot be used for local classifications, for example, constrained to subsamples of lung lobes. Several works to express graph and subgraph similarities are given in the literature [32]. Since we only concentrate on a very basic graph feature we will consider further similarity scores as part of future research.

Mice are valuable models for human diseases in medical research. The analysis of these models is usually done via histology, immunohistochemistry, and biochemical approaches. Therefore topological information is mostly neglected. The development of novel imaging methods like SLOT opened up the possibility to look at the overall architecture of murine lungs and analyse the airway topology with respect to disease, mutations, age, or gender. In addition, SLOT enables the use of fluorescent markers, such as fluorescent labeled antibodies or fluorescence in situ hybridization (FISH) probes, for individual structures of interest which allows correlating lung topology with molecular markers. The results of the exemplary application indicate that the phenotype of SP-D deficient mice which is known to involve alterations at the level of the gas-exchange parenchyma [9–13] is associated with a different airway topology in comparison to wild type animals. The difference occurs on the level of airway branching. More precisely, the feature discriminating the airway topology of these mice is the amount of 4 junctions. However, for a definite correlation further studies of other features are necessary and bigger datasets are required for the training procedure to better account for biological variability. The described method can be extended to further characterize the effect of genetically or environmentally induced lung diseases on airway topology. It provides a simplification of the complex 3D architecture of airway branching and has the potential to discriminate between conserve and variable aspects of lung architecture with respect to disease, mutation, gender, or age specific differences. This might therefore be a valuable tool in lung research using animal models.

## 5. Conclusions

Combining the imaging methods provided by scanning laser optical tomography (SLOT) with digital image analysis, it could be shown that the mouse airway tree can be imaged and analyzed to high degree. As shown, the possibility to determine topology enables classification of exemplary data consisting of wild type and surfactant protein (SP-D) deficient knock-out mice. These results demonstrate that the number of conducting airway generations as well as bifurcation patterns can exhibit specific characteristics relating to illnesses or gene variations.

## Figures and Tables

**Table 1:** Amount of junctions of various degrees found in each group.

Degree	# wild type	# SP-D
4	26	2
5	2	0
6	2	0

#: number of junctions.

**Table 2:** Reclassification error in a leave-one-out experiment, actual classification in columns, leave-one-out result in rows, that is, leave-one-out recognized as wild type, the four wild type samples correctly but additionally also one of the SP-D samples.

Input classified as	Wild type	SP-D
Wild type	4	1
SP-D	0	2
